# Secondary Aortopulmonary Fistula After Thoracic Endovascular Aortic Repair: Case Report

**DOI:** 10.1155/carm/9207891

**Published:** 2026-07-06

**Authors:** María del Mar Conde-Crespo, Miguel Zuñiga, Mauricio Velasquez, Liliana Fernández-Trujillo

**Affiliations:** ^1^ Clinical Research Center, Fundación Valle del Lili, Carrera 98 # 18-49, Cali, 760032, Colombia, valledellili.org; ^2^ Department of Internal Medicine, Hospital Civil de Guadalajara, Salvador de Quevedo y Zubieta No. 750, Guadalajara, Jalisco, Mexico, udg.mx; ^3^ Department of Surgery, Thoracic Surgery Service, Fundación Valle del Lili, Carrera 98 # 18-49, Cali, 760032, Colombia, valledellili.org; ^4^ Faculty of Health Sciences, Universidad Icesi, Calle 18 # 122-135, Cali, 760031, Colombia, icesi.edu.co; ^5^ Department of Internal Medicine, Pulmonology Service, Interventional Pulmonology, Fundación Valle del Lili, Carrera 98 # 18-49, Cali, 760032, Colombia, valledellili.org

**Keywords:** aneurysm, aortic dissection, aortopulmonary fistula, case report, TEVAR, thoracic endovascular aortic repair

## Abstract

Aortopulmonary fistula is a rare but serious condition associated with high mortality, primarily observed in specific patient groups, including those with prior placement of aortic endografts. Its incidence has risen with the adoption of endovascular techniques like thoracic endovascular aortic repair, which, despite its benefits, may lead to complications such as aortobronchial and aortopulmonary fistula formation. However, the current literature offers limited insight into the understanding and management of this condition. Here, we describe the case of a male patient in his late 50s with a history of endovascular repair of chronic Type B dissection of the thoracic aorta, who subsequently developed a complicated aortopulmonary fistula manifesting with recurrent hemoptysis and chest pain. Following extensive evaluations and treatments, the patient underwent open thoracotomy for surgical repair, resulting in a favorable postoperative outcome. Aortopulmonary fistula represents a rare yet significant complication of endovascular procedures. Timely diagnosis and surgical intervention are paramount for enhancing clinical outcomes, although additional research is warranted to enhance our understanding of its pathophysiology and to develop more effective therapeutic approaches. Multidisciplinary collaboration and ongoing follow‐up are imperative for managing this complex clinical condition.

## 1. Introduction

Aortopulmonary fistula is a rare and underdiagnosed condition associated with a high mortality rate of up to 71% [1]. Although aortobronchial and aortopulmonary communications have been reported as a consequence of intrathoracic malignancy, penetrating aortic ulcer (PAU), or aortic trauma, most currently encountered cases occur in the context of a previously placed endoprosthesis [2].

Around the 1990s, with the implementation of thoracic endovascular aortic repair (TEVAR), the incidence of cases of aortobronchial, aortopulmonary, and aortotracheal fistulas increased [3]. This procedure represents a significant technological advancement in the management of aneurysms and thoracic aortic dissection, quickly gaining popularity over open surgery due to its less invasive nature, which reduces morbidity and mortality. However, multiple complications have been reported with its use, including endoleaks, dissection, aortic ulcers, intramural hematomas, and the less common development of fistulization between the aorta and respiratory system structures [3]. Evidence regarding this pathology is scarce, with limited knowledge among medical personnel and the absence of clinical guidelines for timely diagnosis and management.

We present the case of a patient with a history of TEVAR who subsequently developed an aortopulmonary fistula, managed at a high‐complexity institution in Cali, Colombia. The uniqueness of this report lies in the rarity of this complication, which highlights the need to maintain a high index of suspicion and to follow a systematic approach that integrates medical record review and careful interpretation of diagnostic tests to enable early diagnosis.

## 2. Case Report

We present the case of an afro descendant male in his late 50s, with a history of uncontrolled hypertension without adherence to pharmacological therapy and a complication of Type B aortic dissection with a thoracoabdominal aneurysm 14 years ago, managed by TEVAR (Figure 1). Eight years later, open surgery was performed, specifically a superior aortomesenteric bypass and closure of the fenestration of the endoprosthesis, due to disconnection of the two proximal endoprostheses from the thorax. One year later, he presented with a cough and intermittent hemoptysis, for which he visited the emergency department on multiple occasions and finally underwent a chest angio‐computed tomography (CT) scan which reported a previously known endoleak surrounding the origin of the superior mesenteric artery and the celiac trunk, unchanged compared to the previous study, and a new IA‐type endoleak at the fixation site of the two prostheses in the descending aorta (Figure 2).

**FIGURE 1:**
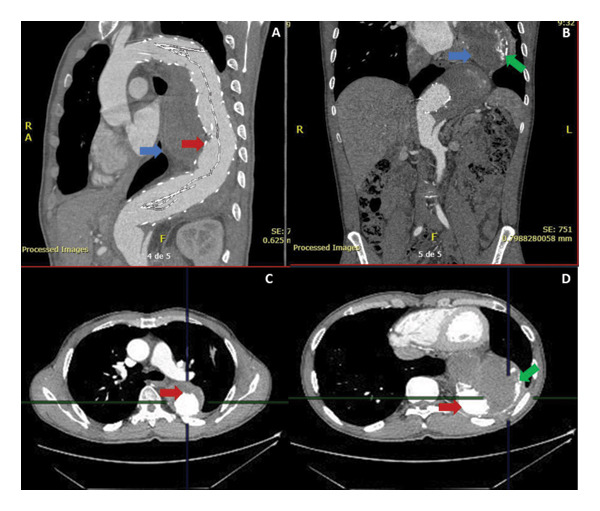
Chest CT scan with coronal views (A, B) and axial views (C, D) showing a thoracic aortic aneurysm (blue arrows) with the endoprosthesis in place (red arrows) and the aneurysmal sac exhibiting thrombosis and calcifications (green arrow).

**FIGURE 2:**
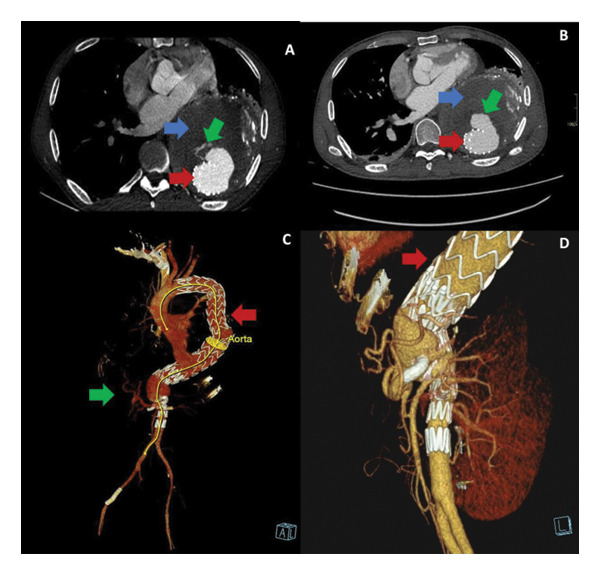
Thoracoabdominal CT angiography with axial views (A, B) and angiographic reconstruction (C, D) showing the aneurysmal sac (blue arrows), the endoprosthesis (red arrows), and an endoleak at the level of the superior mesenteric artery, which is patent and opacifies the false lumen and the aneurysmal sac (green arrows).

Despite the surgical indication for Type I endoleaks, the decision was made for medical management given the patient’s stability. The condition was self‐limited and remained stable for a year, with subsequent consultation for 2 months for a mass sensation in the right hypochondrium and flank with radiation to the dorsal region, burning in nature. An angio‐CT scan was performed, revealing a Type III endoleak (leakage through the endoprosthesis due to rupture, endoprosthesis defect, or failure of seal between prosthetic modules) at the level of the celiac trunk. The leak was identified in the previously implanted Cook TX2 double‐taper endograft (DEZTEG‐2PT‐38‐28‐159‐PF), and the underlying etiology could not be identified. Repair was required with stent placement by TEVAR (Figure 3).

**FIGURE 3:**
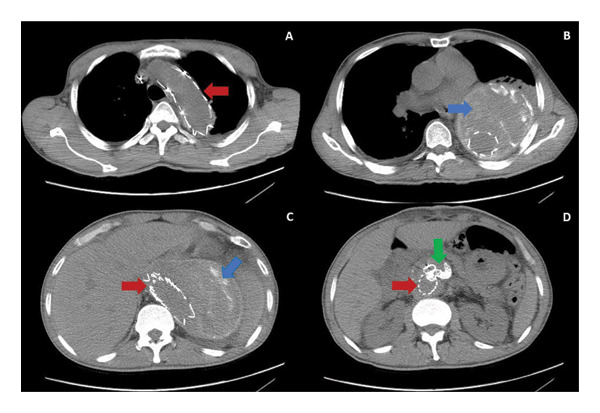
Thoracoabdominal CT angiography showing the aneurysmal sac with a fusiform thrombotic collection and internal calcifications (blue arrows), the endoprosthesis unchanged compared to previous studies (red arrows), and a stent placed in the celiac trunk to manage the endoleak (green arrow).

Three years after the last‐mentioned intervention, following 5 months since the last postsurgical follow‐up with vascular surgery, and under medical management with acetylsalicylic acid 100 mg/day and clopidogrel 75 mg/day for the management of his cardiovascular disease, he presented to the emergency department with insidious sternal pain radiating to the scapular region. The pain was stabbing, exacerbated by postural changes, and accompanied by dyspnea. He denied other associated cardiovascular or respiratory symptoms.

Upon admission physical examination, he was in good general condition, vital signs were within normal limits, and a comprehensive physical examination failed to reveal any pathological abnormalities. Upon admission, an electrocardiogram was performed, which showed sinus rhythm with negative *T* waves in precordial leads V1, V2, V3, and in AVR. Considering the mentioned medical history and clinical presentation, the patient was admitted for suspected aortic dissection.

In the laboratory tests, an ultrasensitive troponin I level of 0.6 ng/mL was found, with a normal range up to 15.6 ng/mL, hemoglobin level of 11.5 g/dL, hematocrit of 37.3%, white blood cell count of 6010/μL, neutrophils at 58.4%, lymphocytes at 23.8%, mean corpuscular volume of 80 fL, platelet count of 119,000/μL, creatinine level of 0.73 mg/dL, C‐reactive protein level of 2.41 mg/dL, prothrombin time (PT) of 11.7 s, International Normalized Ratio (INR) of 1.4, and partial thromboplastin time (PTT) of 31.7 s.

The thoracoabdominal angioscan (Figure 4) described the presence of a periaortic hematoma surrounding the thoracic portion with no changes in size, accompanied by gas bubbles inside, suggesting superinfection or esophageal or bronchial communication, associated with atelectasis in the left lower lobe and alveolar opacities in the right lung field. A fibrobronchoscopy with bronchoalveolar lavage was performed to evaluate the pulmonary infiltrates, revealing changes secondary to extrinsic compression and rotation in the left bronchial tree, with no evidence of active or residual bleeding. Direct examinations and cultures of the lavage were negative, procalcitonin was 0.2 ng/dL, erythrocyte sedimentation rate was 29 mm/h, and blood cultures including fungal cultures were negative, leading to discontinuation of antibiotics initiated upon admission for suspected concomitant infection. Considering the above, it was deemed that the clinical picture and radiological findings could be secondary to the presence of an aortopulmonary fistula; hence, a decision was made to proceed with open thoracotomy for thoracic aortic repair and exploration of the pulmonary component present (Figure 5).

**FIGURE 4:**
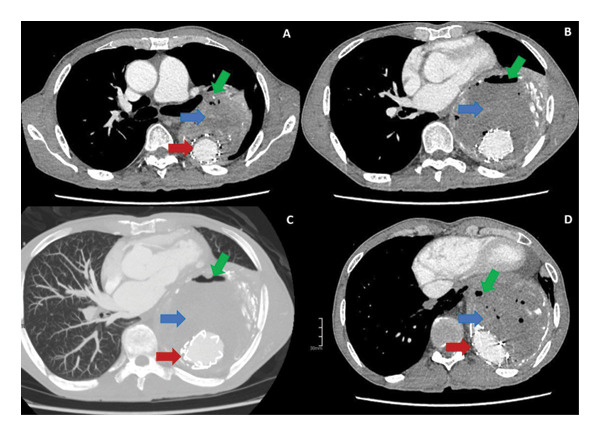
Chest CT scan showing the aneurysmal sac (blue arrows), the endoprosthesis (red arrows), and multiple gas bubbles within the sac, suggesting either infection or esophageal/bronchial communication (green arrows).

**FIGURE 5:**
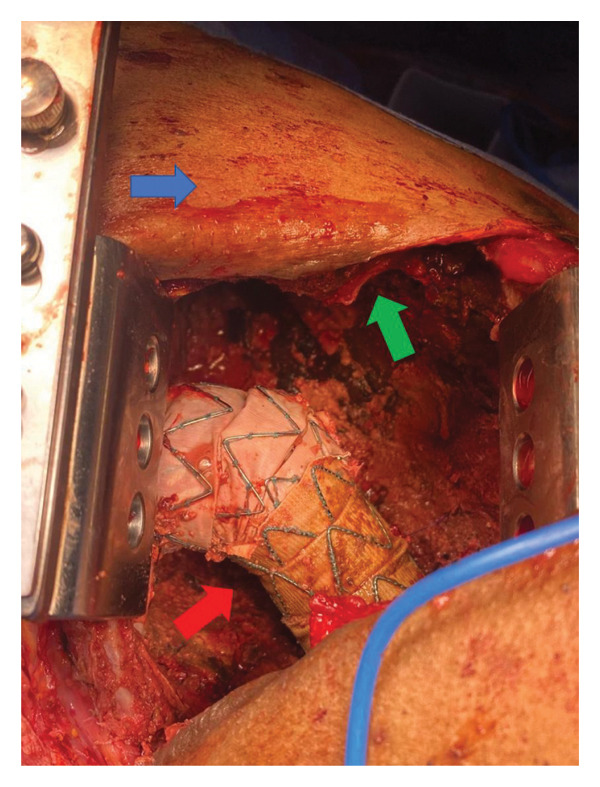
Intraoperative image showing the pleural cavity with the opened aneurysmal aortic sac. The endoprosthesis is visible inside with arterial blood flow from the aorta (red arrow); at the top, the thickened wall of the aneurysmal sac is seen (green arrow), causing collapse of the overlying lung (blue arrow).

During the surgical procedure, a large aneurysmal sac of the thoracic aorta with abundant thrombus content inside was found, along with a communication to the apical segment of the left lower lung lobe. Similarly, adhesion of the lung to the chest wall was observed. The aneurysmal sac was identified and opened, and all the chronic thrombi were removed until the endograft was exposed; the sac was then closed, leaving it folded over the endograft. Additionally, wedge resection of the apical segment of the left lower lobe and decortication were performed. The patient did not experience complications during the procedure. Histopathological examination of the lesion revealed interstitial and subpleural fibrosis and inflammatory infiltration in septa and alveolar spaces, as well as focal hemorrhage, without signs of malignancy.

During the early postoperative period, the patient developed atrial fibrillation with rapid ventricular response, necessitating transfer and management in the intensive care unit with amiodarone 300 mg as a bolus over 1 h, followed by 900 mg to be completed over 24 h via continuous infusion, with a total dose of 1200 mg over 24 h. Additionally, metoprolol 50 mg every 12 h and anticoagulation with enoxaparin 60 mg every 12 h were initiated during hospitalization, with satisfactory evolution.

In the follow‐up chest X‐ray, adequate lung expansion without signs of pneumothorax was identified, along with persistence of left pleural effusion and the presence of an endoprosthesis in the descending aorta. The follow‐up thoracoabdominal angioscan reported the endoprosthesis in a proper position, with permeable visceral stents, and a persistent small Type III endoleak at the level of the celiac trunk stent, managed conservatively and under vascular surgery follow‐up. Infectious disease consultation determined that additional antibiotic therapy was not indicated. With satisfactory evolution, discharge was decided after 7 days of hospitalization, with continued long‐term anticoagulation with apixaban 5 mg every 12 h, along with oral metoprolol tartrate 50 mg every 12 h, furosemide 40 mg tablets daily, and losartan 50 mg tablets daily.

The patient continued under follow‐up with satisfactory progress, evidenced by imaging showing lung expansion without pleural effusion or infiltrates. However, subsequently experienced refractory hemoptysis, necessitating successful embolization of the bronchial artery arising from the right supraclavicular artery. Given the persistence of the clinical picture without additional relevant findings on complementary imaging, the departments of pulmonology, vascular surgery, and thoracic surgery determined that further intervention is not warranted.

For the preparation and publication of this case report, we have de‐identified all the patient’s details. The patient provided written informed consent to undergo all diagnostic and therapeutic procedures described in this report, as well as signed informed consent for publication of the case, including clinical details and accompanying images. Furthermore, the reporting of this study conforms to CARE guidelines [4].

## 3. Discussion

The incidence of aortopulmonary fistulas has been increasing over time, associated with the development of procedures such as TEVAR, whose main indications are thoracic aneurysms, dissections, PAU, trauma, iatrogenic injuries, and aortoesophageal fistulas [5].

In 2015, a study based on the complication’s registry of TEVAR, from the European Association for Cardio‐Thoracic Surgery, was published, including 26 patients. Among these, fistulas were reported, which were aortopulmonary (42%), aortobronchial (54%), and/or tracheal (4%). All these cases had some relevant history such as previous surgical intervention (42%), underlying pathologies like aortic aneurysm (58%), and chronic Type B aortic dissection (15%). The incidence of aortopulmonary fistula found was 0.40 cases per 1000 interventions per year. The average time of appearance of this pathology after TEVAR was calculated at 310 days, with hemoptysis being the main symptom, followed by hematemesis in second place and fever of unknown origin and dyspnea in third place [1].

A retrospective observational study published by Mansukhani et al. describes adverse events associated with the use of TEVAR and defines the most common ones as endoleaks (periprosthetic vascular flow in the aneurysmal sac), procedure‐related dissection, PAU, and intramural hematoma, followed by neurological complications, graft infections, technical device failures, and finally, the least common complication, aortoesophageal and bronchial fistulas in 2.96% of cases [3].

The formation of aortopulmonary fistula is primarily attributed to the aneurysmal dilation of the aorta, which exerts pulsatile pressure on the weakened vascular wall, eventually leading to its rupture into the adjacent organ [6–8]. Additionally, literature suggests that endovascular repair intervention may influence its development, either through endoprosthesis migration or the persistence of lesions such as dissection, ulcer, or hematoma, which over time may rupture and establish communication with the adjacent organ [6, 7].

In the article by Mansukhani et al., the timing of complications was also assessed, with 54.5% occurring late (> 30 days), among which aortoesophageal and/or aortobronchial fistulization can occur up to 3 years after the procedure [3]. This is consistent with the clinical case presented, where the fistula appeared 3 years after the last TEVAR repair, although it should be noted that the patient had undergone another TEVAR 14 years ago, which presented the most described complication (Types I and III endoleaks), requiring a new intervention.

The most common clinical manifestations include intermittent hemoptysis associated with thoracic and/or abdominal pain. A study published in 2015 from Park Hospital in Leipzig, Germany, describes the clinical findings of 374 patients with a history of TEVAR who developed aortoesophageal and bronchial fistulas, where the following were reported in order of highest occurrence: hematemesis, chest pain, hemorrhagic shock, newly developed fever, hemoptysis, melena, dysphagia, vertigo, fatigue, and syncope [9]. It is important to note that some patients may present with mild symptoms or even without symptoms; in fact, it is not uncommon to find patients who only exhibit a limited area of pulmonary atelectasis [9].

One of the major limitations in management is the lack of guidelines for the diagnosis and management of bronchial tree and/or aortopulmonary fistulas. However, a chest X‐ray may reveal a widened mediastinum, tracheal deviation, atelectasis, calcifications, among others. The diagnostic technique of choice is contrast‐enhanced CT and/or angiography, with a diagnostic rate of approximately 89%, allowing visualization of arterial flow using contrast medium. Another diagnostic aid is endoscopy, which has a diagnostic rate of 50% [1, 6, 9].

The management is primarily surgical, and the repair and extent will depend on the damage to the bronchial system or lung parenchyma; it can be performed via open surgery, as in the presented case where thoracotomy with lung decortication and aortic reconstruction was performed, or paradoxically with a new TEVAR intervention, as done in the patient with a Type III endoleak at the celiac trunk level. It is more controversial whether TEVAR is suitable as a long‐term solution, as there are concerns about endoprosthesis contamination and infection, the potential need for lifelong antibiotic suppressive therapy with variable efficacy, and reported complications of reinfection and recurrent bleeding [6, 9, 10].

It has been proposed that definitive treatment involves radical debridement of any portion of the aorta communicating with the lung, aortic reconstruction, and airway repair. In cases where surgical reintervention is not possible, conservative management is pursued, including bleeding control, percutaneous drainage/aspiration with or without TEVAR, and antibiotic management if necessary [6, 9, 10]. Other described causes of aortobronchial and esophageal fistulas include traumatic, infectious, autoimmune diseases, and sarcoidosis, among others [7]; for differential diagnosis, the physician should rely on a detailed medical history and various diagnostic tools [11, 12].

The main limitation of this report is that the information was obtained retrospectively from the medical record, which may have restricted the completeness of clinical details. Nevertheless, given the rarity of aortopulmonary fistula, the present case provides valuable clinical insights that contribute to the existing literature.

## 4. Conclusion

Aortopulmonary fistula presents risk factors such as a history of aneurysms, pseudoaneurysms, or aortic dissection treated with TEVAR. The main symptom is hemoptysis, and the diagnostic tool of choice is CT angiography, which can be complemented with bronchoscopy. The intervention will depend on the extent of the damage. Further advancements in imaging diagnostic technologies and treatment for post‐TEVAR aortopulmonary fistula are needed. Regular analysis of records and the development of management guidelines can help refine diagnosis and select the ideal treatment for each patient.

**Nomenclature**
TEVAR: Thoracic endovascular aortic repair
Angio‐CT: Computed tomography angiography
mg/day: Milligrams per day
mmHg: Millimeters of mercury
ng/mL: Nanograms per milliliter
g/dL: Grams per deciliter
uL: Microliters
fL: Femtoliters
mg/dL: Milligrams per deciliter
PT: Prothrombin time
INR: International Normalized Ratio
PTT: Partial thromboplastin time
ng/dL: Nanograms per deciliter
mL/h: Milliliters per hour
mg: Milligrams
PAU: Penetrating aortic ulcers
CT: Computed tomography

## Author Contributions

María del Mar Conde‐Crespo: literature review, manuscript writing and correction, and final approval of manuscript. Miguel Zuñiga: literature review, manuscript writing and correction, and final approval of manuscript. Mauricio Velasquez: literature review, manuscript writing and correction, and final approval of manuscript. Liliana Fernández‐Trujillo: conception and design, literature review, manuscript writing and correction, and final approval of manuscript.

## Funding

The authors declare that they did not receive financial support for the publication of this manuscript.

## Disclosure

All authors have read and approved the manuscript and significantly contributed to this paper.

## Ethics Statement

This report was prepared in accordance with the ethical standards of the Institutional Ethics Committee and the 1964 Helsinki Declaration. Ethical approval for publication was granted by the Ethics Committee in Biomedical Research of Fundación Valle del Lili, Cali, Colombia (Approval No. 426‐2025, July 28, 2025).

## Consent

Written informed consent was obtained from the patient for publication of this case report and any accompanying images. A copy of the written consent is available for review upon request.

## Conflicts of Interest

The authors declare no conflicts of interest.

## Data Availability

Datasets used and/or analyzed during the current study are available from the corresponding author upon reasonable request.
